# Improved excitation fidelity in cardiac imaging with 2-spoke parallel excitation at 7 Tesla

**DOI:** 10.1186/1532-429X-15-S1-W1

**Published:** 2013-01-30

**Authors:** S Schmitter, L DelaBarre, X Wu, A Greiser, D Wang, EJ Auerbach, J Vaughan, K Ugurbil, P Van de Moortele

**Affiliations:** 1 Center for Magnetic Resonance Res earch, University of Minnesota, Minneapolis, MN, USA; 2 Siemens Healthcare Sector, Erlangen, Bavaria, Germany; 3 Siemens Medical Solutions USA, Inc.; Center for Magnetic Resonance Research, University of Minnesota, Minneapolis, MN, USA

## Background

Cardiac MRI may greatly benefit from ultra high field (UHF) providing higher SNR and intrinsic contrast. But shorter RF wavelength yields transmit B1 (B1+) heterogeneity and contrast variations through the heart. These can be addressed by parallel transmission (pTX) using multi-spoke RF pulses as previously shown in other organs. However, applying pTX in cardiac MRI at 7T is challenging and requires rapid, multi-channel ECG triggered B1+ calibration and ECG synchronized, motion-insensitive pTX acquisitions. In this initial work we investigate the impact of 2-spoke RF pulse design on cardiac imaging at 7T using a 16-channel pTX system

## Methods

Healthy volunteers were imaged at 7T (Siemens, Erlangen) using a 16 channel pTX system with 16 channel transceiver body coil. All sequences were modified to enable both ECG triggering and full pTX capability. B1+ maps of each transmit (TX) channel were obtained using a fast estimation technique [1]. Each map was acquired within 484ms during diastole. 1- and 2-spoke pTX RF pulses (SINC shape, BWTP 4) were designed based on magnitude least squares (MLS) optimization [2] aiming at homogeneous excitation in manually drawn ROI. 3 excitations were compared: a) 1-spoke (= standard excitation) with non-optimized initial RF phase setting (equal amplitude), b) 1-spoke with optimized RF magnitude/phase and c) 2-spoke RF pulse with magnitude/phase optimized for each spoke and channel. To keep a constant RF duration 1-spoke pulses were set to 1600us duration while 2-spoke subpulses were set to 800us. Coefficient of variation (CV, i.e. std/mean) in target ROI, total RF energy and peak energy per channel were computed for all RF pulses for same flip angle. Axial- and 4-chamber views were acquiried using a CINE GRE sequence (resolution 1.2x1.2x5mm3; 1 spoke: TR/TE=41.7/2.5ms, 32 phases; 2 spoke: TR/TE=44.8/2.7ms, 29 phases)

## Results

**Figure 1:**
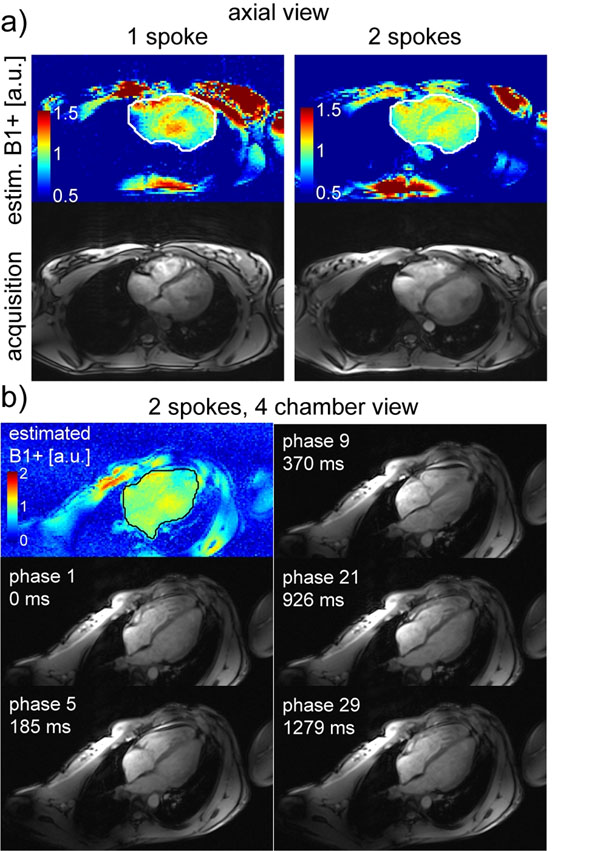
a) estimated B1+ maps for 1 and 2 spoke excitation (upper row) and last cardiac phase of corresponding CINE acquisition (lower row); b) 4 chamber view of different cardiac phases, acquired in CINE mode with 2 spokes at 7T. Upper left corner: corresponding B1+ estimation map (scale different to a) to appreciate overall pattern)

**Figure 2:**
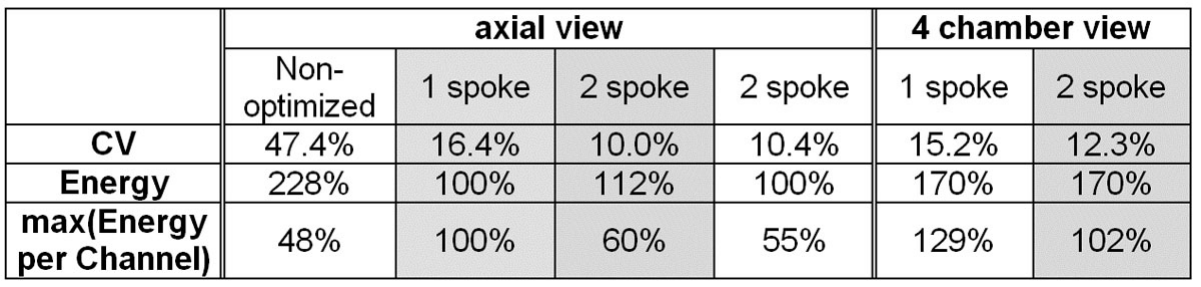
Summarized results of excitation fidelity, RF energy and max. of energy per channel. Gray shaded columns correspond to Fig.1

Fig.1a shows axial views of predicted B1+ maps and GRE cardiac images using 1- and 2-spokes RF pulses. Signal patterns are highly consistent with predictions based on measured B1+ maps, including in more posterior regions (e.g. see aorta; also Fig.1b). Homogeneity improved with 2-spoke, with CV dropping from 16.4% to 10.0% with only minor increase in RF energy (+12% - see Figure 2.1). Fidelity improvement with 2- vs. 1-spoke pulses occured especially close to the ROI edges and posteriorly. Fig. 1b shows GRE images obtained with 2-spoke excitation in 4-chamber view at different cycle phases. 1+2 spokes acquisition in 4 chamber view resulted in higher residual inhomogeneity and higher RF compared to axial views (Figure 2), likely due to larger ROI size and/or orientation. Note the absence of significant excitation pattern change through the cycle.

## Conclusions

pTX 2-spoke excitation in the heart at 7T is feasible, providing improved excitation fidelity, and reduced peak RF amplitude and reduced total RF energy.

## Funding

P41 EB015894, S10RR026783, R21-EB009138, KECK Foundation.
